# Initial testing of a pixelated silicon detector prototype in proton therapy

**DOI:** 10.1002/acm2.12120

**Published:** 2017-07-18

**Authors:** Andrew J. Wroe, Grant McAuley, Anthony V. Teran, Jeannie Wong, Marco Petasecca, Michael Lerch, James M. Slater, Anatoly B. Rozenfeld

**Affiliations:** ^1^ Department of Radiation Medicine Loma Linda University Medical Center Loma Linda CA USA; ^2^ School of Medicine Loma Linda University Loma Linda CA USA; ^3^ Faculty of Medicine Department of Biomedical Imaging University of Malaya Kuala Lumpur Malaysia; ^4^ Centre for Medical Radiation Physics University of Wollongong Wollongong NSW Australia

**Keywords:** proton therapy, radiosurgery, silicon diode radiation detectors, small‐field dosimetry

## Abstract

As technology continues to develop, external beam radiation therapy is being employed, with increased conformity, to treat smaller targets. As this occurs, the dosimetry methods and tools employed to quantify these fields for treatment also have to evolve to provide increased spatial resolution. The team at the University of Wollongong has developed a pixelated silicon detector prototype known as the dose magnifying glass (DMG) for real‐time small‐field metrology. This device has been tested in photon fields and IMRT. The purpose of this work was to conduct the initial performance tests with proton radiation, using beam energies and modulations typically associated with proton radiosurgery. Depth dose and lateral beam profiles were measured and compared with those collected using a PTW parallel‐plate ionization chamber, a PTW proton‐specific dosimetry diode, EBT3 Gafchromic film, and Monte Carlo simulations. Measurements of the depth dose profile yielded good agreement when compared with Monte Carlo, diode and ionization chamber. Bragg peak location was measured accurately by the DMG by scanning along the depth dose profile, and the relative response of the DMG at the center of modulation was within 2.5% of that for the PTW dosimetry diode for all energy and modulation combinations tested. Real‐time beam profile measurements of a 5 mm 127 MeV proton beam also yielded FWHM and FW90 within ±1 channel (0.1 mm) of the Monte Carlo and EBT3 film data across all depths tested. The DMG tested here proved to be a useful device at measuring depth dose profiles in proton therapy with a stable response across the entire proton spread‐out Bragg peak. In addition, the linear array of small sensitive volumes allowed for accurate point and high spatial resolution one‐dimensional profile measurements of small radiation fields in real time to be completed with minimal impact from partial volume averaging.

## 1 INTRODUCTION

As imaging techniques continue to evolve, the targets being presented for treatment in our clinical practice are becoming smaller in size, oftentimes requiring beams of less than 1.0 cm in diameter for treatment. This technical challenge is compounded by expanding treatment sites, including functional radiosurgery, which demand that small beams be delivered with high precision to high doses. These small beam sizes require alternative dosimetry methods including diodes,^1^, ^2^ micro‐ion chambers,^3^, ^4^ and film^5^, ^6^ for measuring beam output, as standard radiotherapy ion chamber devices exhibit partial volume averaging due to their relatively large sensitive volume (SV) size. Protons have the added complication that their Linear Energy Transfer or LET varies as a function of depth (or energy), which can significantly impact detector response. Proton beam scanning and intensity‐modulated proton therapy (IMPT) is another area where accurate and efficient methods for real‐time measurements with high spatial resolution are necessary. In the case of proton beam scanning, not only is an understanding of the machine output at a specific point essential to accurate dose delivery, but accurate beam profile information at various depths in water is also critical for accurate treatment planning and reproducible beam delivery.

For small‐field and beam profile measurements, radiochromic film has often been seen as the standard metrology device, providing high spatial resolution for such applications. However, in the case of proton therapy, radiochromic film can exhibit a varying response to changing LET.^6^ Additionally, radiochromic film also exhibits a number of technical and ease‐of‐use limitations that can limit its deployment in regular clinical QA programs. Chief of these is that postexposure processing limits radiochromic film's ability to provide real‐time data.^7^ In addition, artifacts can be introduced in dose measurements due to properties of the film itself (e.g., variations in active layer or substrate thickness, postexposure intensification), environmental and handling effects (e.g., temperature, light sensitivity), and scanner response (e.g., lateral position artifact,^8^ film orientation,^9^ dust, fingerprints, film curl, and local‐, inter‐ and intrascanner factors).^7^ Finally, inter‐ and intrafilm lot and scanner variation can result in inconsistent dose mapping between film lots, scanner models, and environmental processing conditions.^10^ Recent improvements in technology and techniques, such as multiple‐channel dosimetry,^11^ simplified calibration and intralot recalibration,^10^ calibration‐less relative dosimetry,^12^ and film that is less sensitive to light have significantly mitigated these concerns. They have not, however, eliminated them entirely.

In an effort to identify more efficient and real‐time methods of small‐field dosimetry, the team at the Center for Medical Radiation Physics (CMRP) at the University of Wollongong have continued solid‐state development of small pixelated arrays of monolithic silicon diode detectors. The prototype device tested in this work is referred to as the dose magnifying glass (DMG). It is a pixelated silicon detector that has the potential to provide not only point dose measurements with a high degree of spatial resolution, but also beam profile measurements in real time. However, while this device has been tested in photon therapy, IMRT, and stereotactic radiotherapy,^13^, ^14^, ^15^ it has yet to be tested in proton therapy. Accordingly, the uniformity of response across the proton spread‐out Bragg peak (SOBP) was unclear.

The goal of this work was to evaluate a DMG prototype in proton fields typically associated with radiosurgical applications at Loma Linda University Medical Center (LLUMC). The response of the device was compared to a commercially available PTW proton diode, a PTW plane‐parallel ionization chamber, and Gafchromic EBT3 film. Additionally, an in‐house developed and validated Geant4‐based Monte Carlo application was used for comparison. Both depth dose and lateral profiles were evaluated. It was hypothesized that these tests would not only evaluate the DMG against established forms of metrology, but also identify future directions for development.

## 2 METHODS

**Figure 1:**
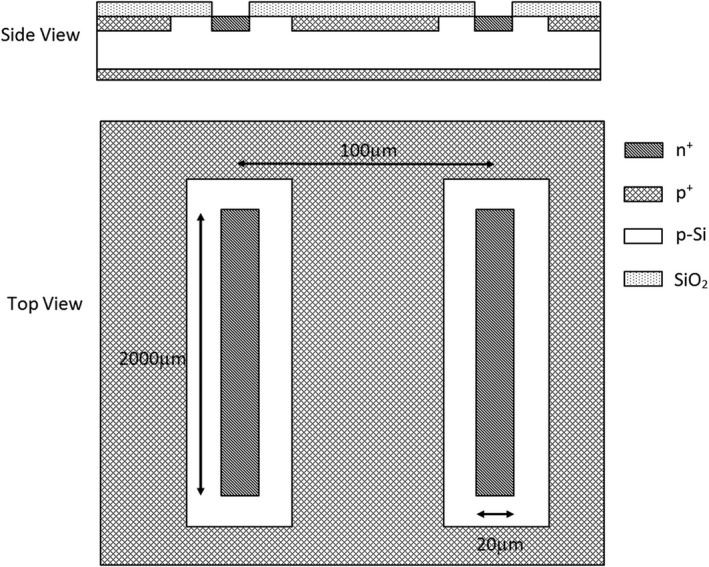
Schematic of two n+ SV elements in the DMG detector. Note the entire DMG is made up of 128 SV elements.

The DMG is an array of 128 n+‐strips of 2 mm length and 20 μm width on a p‐type silicon substrate 380 μm thick. The pitch (or SV center‐to‐center separation) is 100 μm with a p‐stop implantation between the microstrips (80 μm) for compensation of the accumulation layer generated by irradiation of the thick silicon dioxide.^13^, ^14^, ^15^ A schematic of the DMG SV array is displayed in Fig. 1. The assembly was pre‐irradiated to a dose of 4 MRad with Co‐60 before deployment for testing.

**Figure 2:**
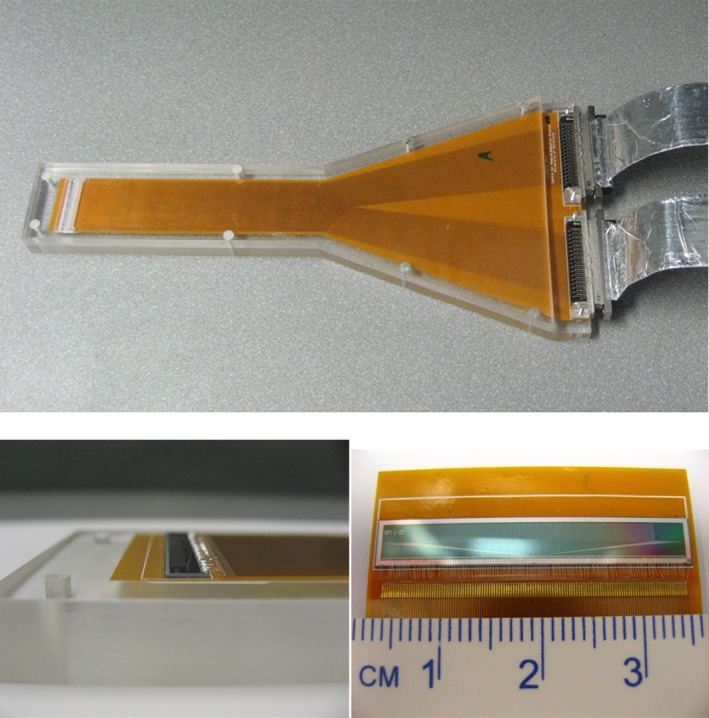
Images of the DMG probe used in this work. The minimal thickness (lower left) allows for a wide range of mounting options and minimal perturbation of the proton beam while the linear array (lower right) allows for concurrent point dose and beam profile measurements.

The DMG is glued and wire bonded onto 400 μm thick kapton carrier to provide the proper fan out of the signal for connecting the readout electronics and minimize the impact of these connections on the proton scatter conditions. The detector is used in passive mode (no bias applied at the contacts) and readout is carried out with the detector configured as a planar detector with a common electrode p+ from the same side as the n+ strips. For this work, the DMG was mounted to a Lucite probe holder (Fig. 2) for rigidity and to facilitate mounting in a water tank; however, the compact nature of the device allows for a wide range of mounting options, including within specialized phantoms and probes.

**Figure 3:**
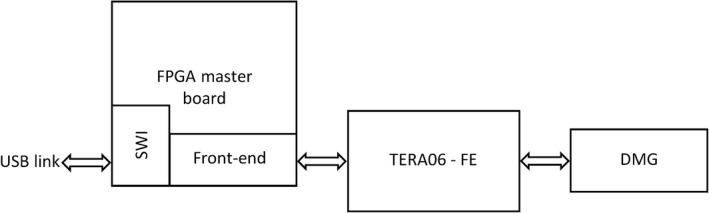
DMG data acquisition architecture.

Figure 3 shows the schematic block diagram of the DMG data acquisition system. The FPGA motherboard houses a Xilinx XC3S400 along with a Cypress USB interface to establish the communication with the host computer. The motherboard drives the digital interface of the TERA06 board, a dual 64 channel preamplifier. The two TERA06 chips read out the 128 detector channels simultaneously.^16^ Each channel of the TERA06 is a charge‐to‐frequency converter equipped with a 16‐bit counter that records the number of times the input charge (accumulated into a capacitor during the integration time) exceeds the quantum charge, settable by an analog potentiometer. A digital reset is used for zeroing the counter values immediately after each frame is acquired. A graphical user interface (GUI) has been custom designed at the CMRP and provides the operator with all the controls to acquire the data from the detector in real time and also perform preprocessing of the raw binary data.

The GUI is compiled under the C++ developing suite Nokia QT rev4.0. It manages the USB link using dynamic language libraries (DLL) specifically developed for the USB Cypress interface. It initializes the COM‐link, sends the firmware to the FPGA, and acknowledges if the device is connected and fully operational. The operator controls the acquisition settings and the relevant parameters such as the integration time, the total acquisition time, and all the complementary signals necessary for the operation of each TERA06 chip. The motherboard stores the data into an internal FIFO (First‐In First‐Out) and triggers the GUI to download the data in burst‐mode. The size of the burst can be user‐defined and optimized based on the CPU computation load of the host computer to minimize the USB latency, which varies between 2 and 3 ms. Data losses are avoided through the use of a dual, cascade FIFO buffer stage of 17 Kbyte of RAM.

**Figure 4:**
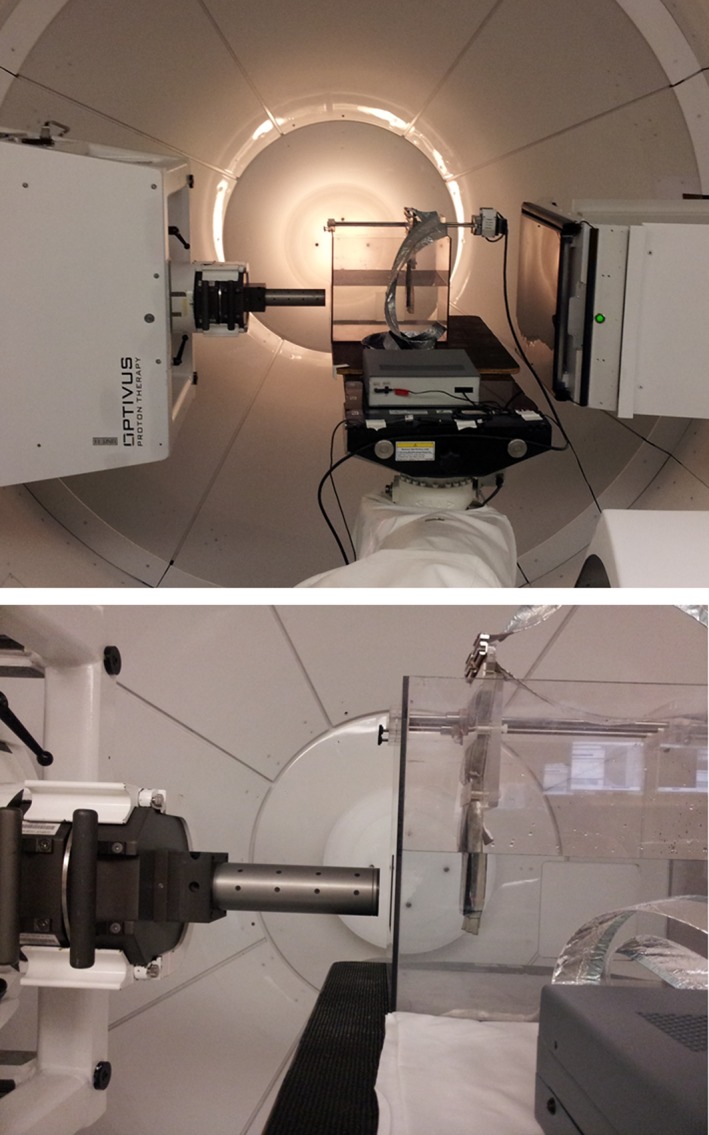
DMG experimental setup for depth dose and beam profile measurements with a magnified view (bottom). Note the extended X‐ray imager (top) which is used to ensure detector alignment and parallel travel with the beam central axis.

Testing of the DMG prototype was conducted in the Gantry 1 treatment room at LLUMC. This treatment room is fitted with a 6‐degree‐of‐freedom robotic patient positioner and associated 2D orthogonal imaging system^17^ to aid in alignment of the patient and, in this case, the experimental setup (Fig. 4). Depth dose and lateral beam profiles were measured for our standard radiosurgery energies of 127 MeV (90% dose range of 9.88 cm) and 157 MeV (90% dose range of 15.15 cm) through a single‐stage scattering system. The single‐stage scattering system provides a maximum field size of 4 cm diameter with collimation provided via a dedicated radiosurgery cone that attaches to the end of the proton nozzle.^18^ This system allows for minimal air gap between the collimator and the patient, ensuring that the proton penumbra is minimized while maintaining a high dose rate. Field sizes measured and presented here were 0.5 and 2.0 cm in diameter, created by brass inserts 7.5 cm in thickness along the beam axis. An array of beam modulations were tested that are typical for radiosurgical applications, including an unmodulated case that is representative of what can be used in functional radiosurgery or proton beam scanning treatments.

Depth dose and lateral profiles were measured in a water tank measuring 250 mm × 250 mm × 500 mm with a 180 mm diameter Lexan window 6 mm thick at the upstream surface. Detector motion was facilitated by a linear corkscrew rail and stepper motor, which can be orientated either parallel or perpendicular to the beams central axis. The stepper motor is controlled with a single‐axis programmable controller and micro‐step driver with USB 2.0/RS‐485/Modbus‐RTU communication, and user interface is achieved via custom designed LabVIEW 2011 software (National Instruments Corporation, Austin TX, USA). The DMG was waterproofed for this work through the use of a custom plastic cover, which also served to make the device light proof. For comparison with the DMG, depth dose information for the 2 cm diameter beam was also collected with a PTW PR60020 proton diode and a PTW Markus N23343 plane‐parallel ion chamber. These detectors were selected as the PTW PR60020's stability of performance in proton therapy has been demonstrated previously,^1^ while the PTW Markus has been in continued clinical use at our facility for over 25 yr. The PTW Markus N23343 is a plane‐parallel ionization chamber with a SV of 0.055 cm^3^ vented to air and a 0.03 mm polyethylene entrance window. The device was used with a water‐equivalent and water‐proof protective cap of 1.06 mm for in water measurements. The PTW PR60020 proton diode has a single cylindrical SV with a cross‐sectional area of 1 mm^2^ and a thickness of 20 μm, located behind a 1.33 mm water‐equivalent window.

For beam profile measurements using Gafchromic film, 9 × 5 cm pieces of Gafchromic EBT3 film (Advanced Materials Group, Wayne, NJ, USA) were placed in custom designed holders and positioned at a specified depth within a water tank. Films were irradiated using unmodulated beams at the same energies (i.e., 127 and 157 MeV), water‐equivalent depths (WED), and treatment nozzle configurations as analogous DMG and MC data. Irradiation duration for film measurements was ~3 or ~3.33 min for 127 and 157 MeV, respectively. Films were scanned in 48 bit RGB format at 72 dpi using an Epson 10000XL flatbed scanner. Dose maps were created with Film QA Pro software (Ashland Inc., Wayne, NJ, USA) using a triple‐channel dosimetry method based on a rational function‐fit to previously exposed calibration films.^11^ Cross‐sectional profiles of the dose maps were obtained using Film QA Pro. Data were normalized based on the maximum dose value.

At LLUMC, protons are accelerated by a synchrotron and delivered to treatment rooms in 2.2 s pulses. Thus, the DMG records signal from impinging protons separated by time intervals where the device is in a quiescent state. Therefore, before analysis, quiescent background time points were separated from data time points and the average background signal was subtracted from each data time point. Depth dose profiles were determined using channels 85–115 (which corresponds to a 3.1 mm width of sensitive area perpendicular to the beam) and normalized to data measured by the DMG at the shallowest WED for each experimental case (i.e., 48, 50, or 68 mm WED). Two additional steps were applied when processing transverse profile data: a calibration step and removal of over/under‐responsive channels. Calibration factors for each channel were calculated for each energy (i.e., 127 and 157 MeV) at the level of the unmodulated Bragg peak for a 2 cm diameter proton field. Because the 2 cm beams have a flat profile over the lateral extent of the DMG, all channels were normalized to the same constant signal and the corresponding calibration factors were applied to each channel. Certain channels in the prototype device did not produce a signal, or were excessively over/under‐responsive. These later channels (so designated if the absolute value of their signal had a percent difference greater than 50% when compared to the average of four neighboring nonzero channels) were replaced with zero signal. Transverse dose profiles were normalized with respect to the profile centroid.

**Figure 5:**
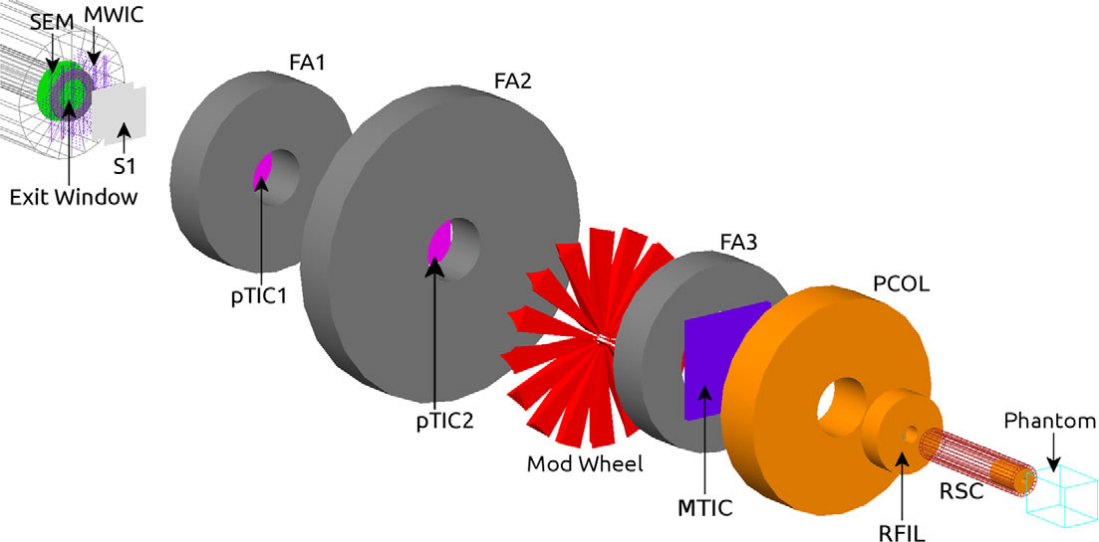
Schematic diagram of the simulated geometry of the Gantry 1 treatment nozzle with radiosurgery cone geometry used in Geant4 Monte Carlo simulations. The components are: the secondary emission monitor (SEM) and titanium exit window, multiwire ion chamber (MWIC), initial scatterer (two lead wedges) (S1), first proton transmission ionization chamber (pTIC1), first steel fixed aperture (FA1), second pTIC2, second fixed aperture (FA2), Perspex modulation wheel, third fixed aperture (FA3), multielement transmission ion chamber (MTIC), brass precollimator (PCOL), precollimator and ridge filter (RFIL), radiosurgery cone (RSC), and water phantom.

Monte Carlo simulations provided further comparative data for both depth dose and lateral profiles. These simulations were performed using software developed in‐house that incorporates the Geant4 toolkit.^19^, ^20^ Protons and secondary particles were tracked through a model of our Gantry 1 treatment nozzle^21^, ^22^ using a custom physics list incorporating: low energy Livermore physics models for electromagnetic interactions, binary cascade models of inelastic interactions of hadrons and heavy ions, and the high‐precision neutron package.^23^ The treatment nozzle configuration and proton energies were chosen to mimic the experimental conditions described above (Fig. 5). In particular, simulated 127 or 157 MeV protons were delivered through a single‐stage scattering system to a voxelized water phantom. The beams were either unmodulated, or modulated to 15, 30 or 60 mm. Total dose was scored in each voxel, and depth dose and transverse profiles were calculated using software developed in Python. Depth dose profiles for 20 mm diameter beams were determined using voxels of 1.0 × 1.0 × 1.0 mm, phantom range cuts of 250 μm, and 1.3 billion particle histories per simulation. Modulated depth profiles were normalized with respect to dose at the center of modulation (COM) of the spread‐out Bragg peak, and unmodulated depth dose profiles were normalized by maximum dose. Depth dose profiles were determined using the central array of 2 × 2 × 1 voxels column of voxels (thus presenting a sensitive area of 2 × 2 × 1 mm^3^ for depth dose analysis). Transverse dose profiles of 5 mm diameter beams were determined using voxels of the same size as the active element of the DMG (X × Y × Z dimensions of 0.02 × 2.0 × 0.38 mm with a center‐to‐center element spacing of 0.1 mm) with range cuts of 100 μm and 5.1 billion histories. Transverse profiles were normalized with respect to maximum dose.

## 3 RESULTS

**Figure 6:**
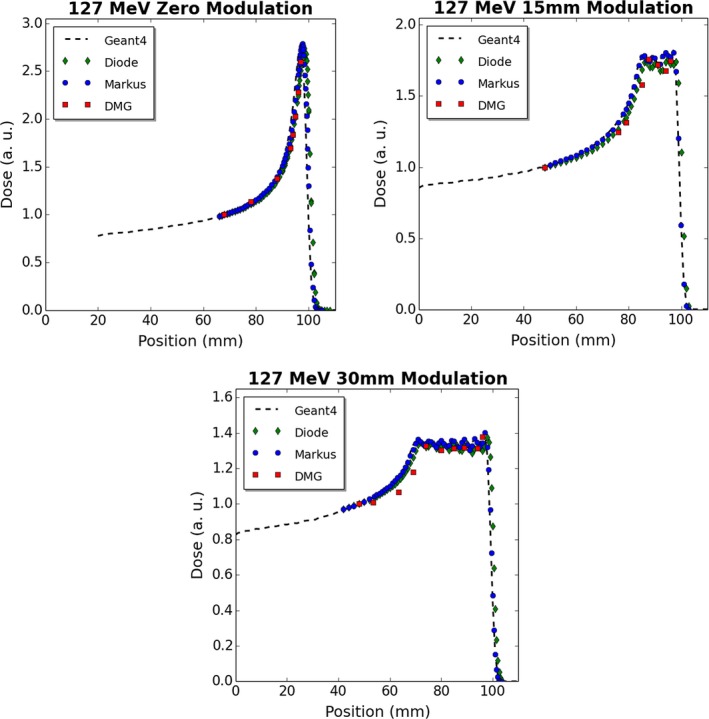
Measured and simulated depth dose profiles for varying beam modulations in water for a 127 MeV, 20 mm diameter proton beam.

**Figure 7:**
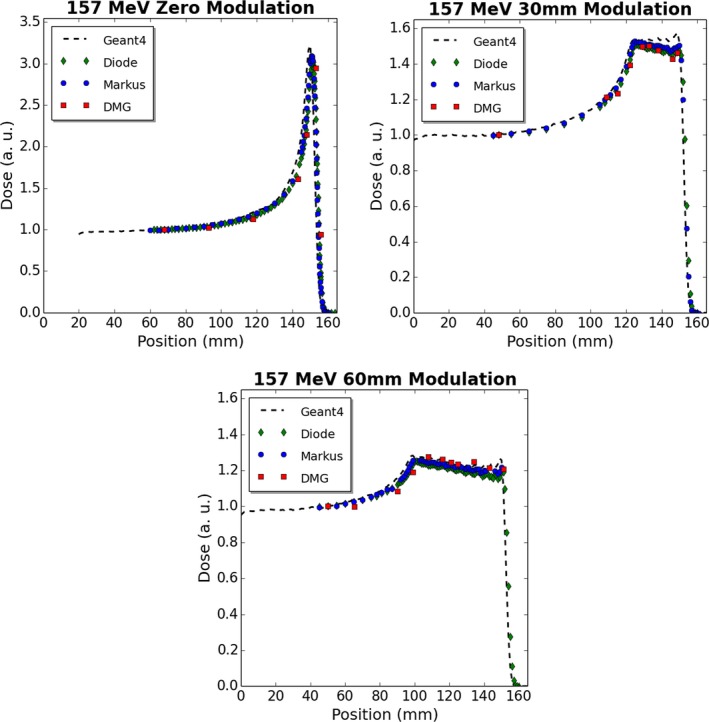
Measured and simulated depth dose profiles for varying beam modulations in water for a 157 MeV, 20 mm diameter proton beam.

The DMG can be operated as a simple dosimeter, which charge measured over either a single SV or multiple SV's across a given linear displacement. The single SV option allows for point measurements of a high spatial resolution (2 mm long and 20 μm wide) to be made, which is of significant benefit in very small or high‐gradient radiation fields. The disadvantage of this mode of operation is that the small SV requires longer acquisition times to accrue sufficient statistics. It is envisaged that the DMG would be operated more typically as a single array of SV's which all individually contribute charge‐to‐detector response at a given location. The number of SV's analyzed is customizable and provides the user greatest flexibility with selecting a total SV size suitable for the field size under study. This later mode was chosen to evaluate the DMG performance in measuring the depth dose profiles of a passively scattered proton beam (Figs. 6 and 7). Thirty DMG SV elements were used at each measurement depth (corresponding to a total SV length of 3.1 mm perpendicular to the beam) of a 127 MeV or 157 MeV 20 mm diameter proton beam, for comparison with a commercial PTW diode, PTW Markus parallel‐plate ionization chamber and Monte Carlo.

For unmodulated proton beams, the proximal edge of the Bragg peak and the peak location was measured accurately by the DMG. Modulated data measured by the DMG exhibited good agreement to the PTW diode and Markus chamber, especially across the SOBP. Across the plateau region of the SOBP, the DMG exhibited an average response within 1% of the PTW diode, for all cases studied except 157 MeV with a 60 mm modulation. For the 157 MeV 60 mm modulation case, the shape of the SOBP was well represented by the DMG, however the DMG did over‐respond in the SOBP region by an average of 3.3% when compared to the PTW proton diode and an average of 1.4% when compared with the PTW Markus plane‐parallel ion chamber.

As all depth dose data were normalized at the depth of shallowest DMG measurement, analyzing the COM response of each detector type relative to a commercially available small SV solid‐state detector (PTW diode) with a demonstrated stability in proton therapy,^1^ provides an indication of detector stability in this variable LET region. These data, displayed in Table 1, demonstrated that the relative response of the DMG at the COM was within 2.5% of a PTW diode detector for all energy and modulation combinations tested. Additionally, the DMG data do not show a decrease in response over the SOBP region and the shape of the SOBP compares well with the other detector modalities. Both of these features indicate an LET independence of this device for clinical proton therapy energies, albeit further measurements may be necessary with discrete proton energies to further validate this.

**Table 1:** Relative dose measured by each modality at the COM for varying beam energy and modulation combinations. The difference (as a percentage) of each modality is calculated relative to the response of the PTW Diode and represented as a percentage (Difference = 100 × (D_x_‐D_diode_)/D_diode_)

	Relative dose at COM	Difference wrt PTW Diode (%)
127 MeV–15 mm modulation		
DMG	1.720	0.175
PTW–Diode	1.717	–
PTW–Markus	1.725	0.446
Geant4	1.731	0.821
127 MeV–30 mm modulation		
DMG	1.314	−1.754
PTW–Diode	1.338	–
PT0057–Markus	1.355	1.268
Geant4	1.333	−0.353
157 MeV–30 mm modulation		
DMG	1.475	−0.860
PTW–Diode	1.487	–
PTW–Markus	1.504	1.114
Geant4	1.545	3.857
157 MeV–60 mm modulation		
DMG	1.233	2.465
PTW–Diode	1.203	–
PTW–Markus	1.222	1.581
Geant4	1.228	2.044

**Figure 8:**
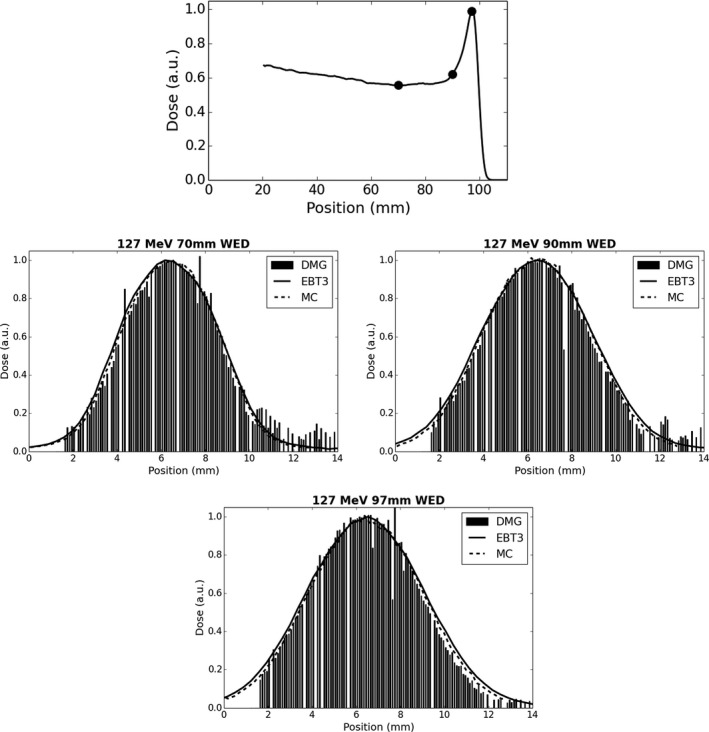
Measured beam profiles at varying depths in water for a 127 MeV proton beam. Geant4 simulation data are also provided for comparison. A simulated central beam axis depth dose profile utilizing 0.25 × 0.25 × 0.25 mm^3^ voxels with DMG measurement locations marked is provided for reference (top).

**Figure 9:**
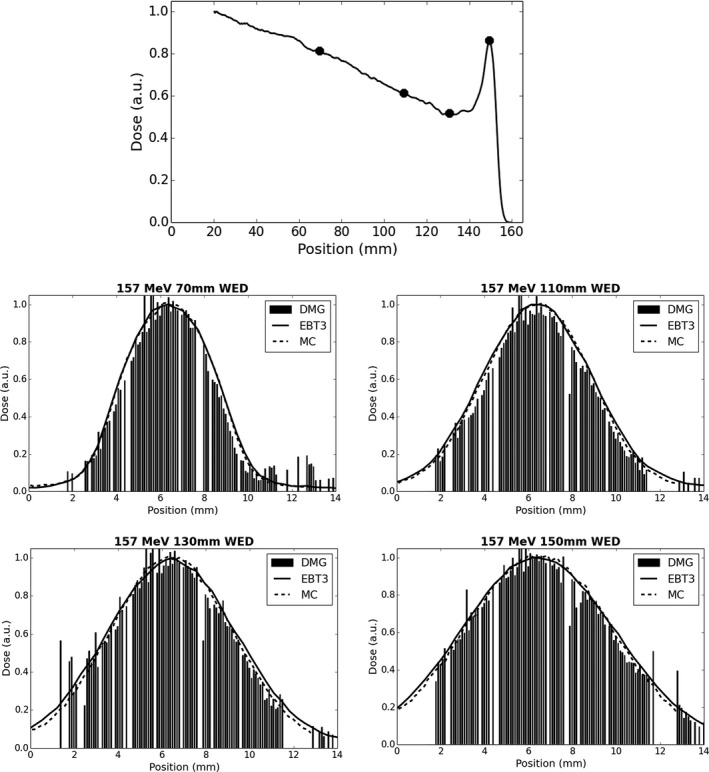
Measured and simulated beam profiles at varying depths in water for a 157 MeV proton beam. Geant4 simulation data are also provided for comparison. A simulated central beam axis depth dose profile utilizing 0.25 × 0.25 × 0.25 mm^3^ voxels with DMG measurement locations marked is provided for reference (top).

In routine QA applications, it is expected that the DMG will be operated using all SV elements to gain both point dose measurement data as well as beam profile data. Data were collected for 5 mm diameter, 127 and 157 MeV proton beams at various depths along the depth dose profile (Figs. 8 and 9). Excellent agreement in profile shape was observed for all measurement locations when compared with Monte Carlo and EBT3 film data. Note that the edge of the detector array is at a displacement of 1.6 mm and 1.7 mm for the 127 MeV and 157 MeV cases respectively, so no data are available below these displacements. For 127 MeV, the FWHM and FW90 reported by the DMG was within ±1 channel (0.1 mm) of the Monte Carlo and EBT3 film data across all depths tested. The 157 MeV data exhibited additional statistical uncertainty in the measurement leading to the suppression of additional channels (see Method) across the measured profile. These suppressed channels are clearly visible as null channels in Figs. 8 and 9. On average between displacements of 2 mm and 11 mm, there was on average eight suppressed channels for the 127 MeV case while for 157 MeV profiles the number of suppressed channels increased to 14. While the additional null channels for the 157 MeV case do impact the total data acquired, it did not impact agreement of the overall beam profile shape as the FWHM and FW90 were measured by the DMG to within ±2 channels (0.2 mm) of the Monte Carlo and EBT3 film data for all depths tested.

## 4 DISCUSSION

The beam profiles measured with the DMG showed excellent agreement with Monte Carlo and Gafchromic film data. The high spatial resolution of the device is comparable to that of film and allows for measurement of the smallest fields currently used in clinical proton therapy (i.e., 5 mm diameter). Unlike film, that requires postprocessing and special handling, the DMG provides readout that is refreshed in real time via a graphical user interface. The real‐time nature of the data acquisition is very useful, especially for proton pencil beam scanning applications, where efficient real‐time feedback to accelerator staff is important during the acceptance testing and commissioning process. When comparing with other data sets or if further analysis is required, the data can be output for graphing and analysis using any of the common computer‐based tools for this purpose.

The DMG tested in this project is a prototype instrument and certain refinements are necessary prior to clinical deployment in proton therapy. Firstly, several channels were removed from analysis because they either were unresponsive or produced excessively high amounts of electronic noise that could not be corrected for. Such channels may be caused by issues in the manufacturing process and may be exacerbated by sensitive cabling and connections. The loss of data can impact measured results and lead to the generation of an incomplete data set, however, in this study the suppression of these channels in our prototype did not significantly impact the beam profile agreement with either Monte Carlo or EBT3 film. In addition, for production DMG detectors, scanning laser systems such as those used in high energy physics^24^ could be used to evaluate the DMG systems prior to deployment allowing for selection of units that have all of their SV elements operating within acceptable limits. Beam profile measurements for 157 MeV protons exhibited increased noise and statistical uncertainty, which can be traced to the cabling system in this existing prototype. The aluminized ribbon cables between the DMG probe and the DAQ system proved to be very delicate and underwent degradation as the experiments progressed. The ruggedness of this will have to be addressed as we move toward a clinical system.

When applying a detector system to scanned proton therapy, the detector's ability to handle high instantaneous dose rates needs to be considered. In this study, the DMG was tested in a pulsed proton therapy environment provided by a synchrotron with a 0.4 s pulse duration and a 2.2 s duty cycle.^25^ Average dose rates of up to 12 Gy/min, corresponding to an instantaneous dose rate of 66 Gy/min, were tested with stable results. Above this value, some under response of up to 10% was observed for average dose rates of 60 Gy/min. While these dose rates are rarely seen in passively delivered clinical proton therapy applications, they can be encountered in proton beam scanning applications. Additionally, as the trend of radiation treatment has been toward higher dose rates and faster treatments, especially when applied to SRS and SBRT treatments, it can be foreseen that a uniform response at higher dose rates would further widen the clinical application of the DMG. An improvement in this regard is already under investigation, as we believe that uniform response with dose rates exceeding 12 Gy/min (average) may be achieved by reducing the integration time of the system. By doing so, it is hypothesized that counts will not be missed when the collected charge is too high (i.e., in a high dose‐rate environment).

The DMG prototype provided a small linear array of 2000 μm × 20 μm × 380 μm SV's for measurement of beam profile data and dose with high spatial resolution. The ability to measure both parameters at a given depth or for a given experimental setup in air is certainly more efficient than using a scanned single volume detector or film which requires postprocessing and does not provide information in real time. The high spatial resolution and small SV size of the DMG does mean that data acquisition times are longer, requiring 5–10 Gy per measurement to achieve adequate statistics. However, extended data acquisition times for small SV detectors is a suitable tradeoff given the spatial resolution enhancements they provide. Further reduction of the SV size below 2000 μm in one dimension would further improve the spatial resolution of the device and reduce any impact of partial volume averaging for very small fields, although this would be at the cost of data acquisition times.

The size of the DMG array and the one‐dimensional nature limit the clinical applications to very small proton fields if beam profile measurements are required. Increasing the size of the array would widen its application to larger passively scattered proton fields and also to lower energies in proton pencil beam scanning applications, which typically have wider beam spots and longer low dose tails. The addition of either a second orthogonal array of SV's or a complete 2D array of SV's would also allow for concurrent measurement of point dose and orthogonal beam profiles (or the entire beam profile in the case of a 2D array), which would provide further QA information in pencil beam scanning applications where the size and shape of the proton beam also need to be evaluated. The feasibility of this approach was recently demonstrated by the CMRP in development, characterization, and application of a 2D monolithic silicon pixel array (with submillimeter SVs and a pitch of 2 mm) in dose mapping on a medical linac.^26^, ^27^

The DMG tested here has demonstrated that it can be used to provide real‐time measurements of both point dose and beam profiles of pencil proton beams with up to 0.02 mm resolution. Such resolution is key in obtaining a precise picture of the sharp dose gradients when the detector is properly orientated with the radiation field. This information, while very useful for collimated beams used in proton radiosurgery and functional radiosurgery, is also especially useful in pencil beam scanning applications for both commissioning and routine QA. During the commissioning process, the collection of accurate beam profile data as a function of depth in water for the complete clinical range of proton energies is essential for evaluation of system performance/stability and also for input to the treatment planning system (or validation of a Monte Carlo system which generates data for the treatment planning system^28^). Such measurements are typically completed using radiochromic film and/or scanned ion chamber detectors;^28^, ^29^ however, a pixelated silicon detector such as the DMG may provide a means for measuring these profiles in real time with greater efficiency as profile data can be measured at each depth without the need for lateral detector scanning. Additionally, for daily QA of pencil beam proton systems, many centers use ion chamber arrays such as the I'mRT MatriXX (IBA Dosimetry, Schwarzenbruck, Germany, model I'mRT MatriXX) which comprise a of 2D array of 32 × 32 ion chambers with a center‐to‐center spacing of 7.6 mm and a SV size of 4 mm diameter and 5 mm height. The DMG may help augment these systems, especially if it can be developed as a nozzle‐mounted 2D pixelated transmission sensor system.^30^ The improved spatial resolution of the DMG would allow for more accurate daily analysis of the proton beam size and shape for evaluation of accelerator/beam transport performance.

## 5 CONCLUSION

The DMG prototype tested here proved to be a useful device at measuring depth dose profiles in proton therapy, with negligible variation in response across the clinical proton SOBP. In addition, the small SV allowed for accurate point measurements of small radiation fields to be completed without the partial volume averaging exhibited by larger ion chamber detectors. The device's high spatial resolution and linear SV arrangement also allowed for beam profiles to be measured concurrently with depth dose measurements in real time. The dual function of this device lends itself to QA measurements in small‐field proton radiosurgery and also to beam commissioning in proton beam scanning, where accurate and efficient measurements of the pencil beam profiles as a function of depth are of paramount importance to intensity‐modulated proton therapy treatment planning. The application of 2D silicon pixel transmission arrays based on this technology, and already developed by the CMRP, could be the next step in real‐time QA and commissioning in proton therapy.
